# A Comprehensive View on the Mechanisms of Coronavirus Escaping Innate Immunity

**DOI:** 10.3390/vetsci12121156

**Published:** 2025-12-03

**Authors:** Yichen Liu, Hao Lu, Jinyuan Li, Yuqing Xie, Gaowei Hu, Shengmei Pang, Siqi Lian, Jiaqi Liu, Guoqiang Zhu, Xueyan Ding

**Affiliations:** 1 College of Veterinary Medicine, Henan Agricultural University, Zhengzhou 450046, China; 2 Molecular Biology Laboratory, Zhengzhou Normal University, Zhengzhou 450044, China; 3 College of Life Sciences, Taizhou University, Taizhou 318000, China; 4 College of Veterinary Medicine, Yangzhou University, Yangzhou 225009, China; 5 Henan Province Key Laboratory of Animal Food Pathogens Surveillance, Zhengzhou 450046, China; 6 Key Laboratory for Animal Pathogens and Biosafety, Ministry of Education, Zhengzhou 450046, China

**Keywords:** coronaviruses, innate immunity, antiviral mechanisms, immune escape mechanisms

## Abstract

Coronaviruses are a group of widespread infectious pathogens that pose a serious threat to human and animal health. Corona Virus Disease 2019, instigated by severe acute respiratory SARS-CoV-2, has presented an unprecedented challenge to global public health in recent years. The host innate immune system is the first line of defense against pathogens, which plays a key role in inhibiting the initial infection of viruses and regulating the initiation and development of acquired immunity. However, coronaviruses can suppress the host’s innate immune response through their unique immune escape mechanisms, which is one of the key strategies for coronavirus pathogenesis. This review focuses on the host’s innate immune sensors, innate antiviral immune responses, and the mechanisms employed by coronaviruses to evade and suppress the innate immune system. And we hope that it will contribute to a comprehensive understanding of the escape mechanism of coronaviruses regarding innate immunity and the pathogenesis of coronaviruses.

## Simple Summary

Coronaviruses, including severe acute respiratory syndrome coronavirus 2, employ sophisticated strategies to evade the host innate immune system, which serves as the first line of defense against viral infections. These pathogens suppress antiviral responses by encoding proteins that interfere with pattern recognition receptors, inhibit mitochondrial antiviral signaling function, and disrupt interferon (IFN) production and signaling. Key mechanisms include downregulation of major histocompatibility complex class I molecules, conformational shielding of the receptor-binding domain to avoid immune detection, and exploitation of host proteases for viral entry. Additionally, non-structural proteins and accessory proteins (e.g., ORF6, ORF8) antagonize IFN pathways and impede nuclear translocation of immune signaling molecules. Understanding these escape mechanisms is critical for developing broad-spectrum antiviral therapies and vaccines that target conserved viral elements, enhancing our ability to combat coronavirus infections and mitigate future outbreaks.

## 1. Introduction

Coronavirus, known for its membrane and corona-like surface projections, is a single-stranded plus-stranded RNA virus divided into four genera, α, β, γ, and δ, according to genomic structure and serotype. Among them, β coronaviruses, such as severe acute respiratory syndrome coronavirus 2 (SARS-CoV-2), severe acute respiratory syndrome coronavirus (SARS-CoV) and Middle East respiratory syndrome coronavirus (MERS-CoV), pose a major threat to global health due to their significant infectivity and pathogenicity [1]. SARS-CoV-2 and SARS-CoV invade host ciliated bronchial epithelial cells and alveolar epithelial type II cells (AT2) by binding their S-spike proteins to angiotensin-converting enzyme 2 (ACE2) receptors on the surface of host cells [2,3], while MERS-CoV invades the body by using dipeptidyl peptidase 4 receptors [4]. Due to amino acid differences in the receptor-binding domain (RBD) of S protein, the binding force between SARS-CoV-2 and ACE2 receptor is much higher than that of SARS-CoV, which may be the key to its high infectivity [5]. It was also found that SARS-CoV-2 also used the transmembrane serine protease 2 (TMPRSS2) to enhance the binding to ACE2 receptors to improve the invasion efficiency [6].

From the perspective of zoonotic diseases, both alpha- and beta- coronavirus genera rely on the wide recognition ability for conserved receptors such as ACE2 and aminopeptidase N, entering human populations via intermediate hosts like bats, rodents, camels, raccoon dogs, and civets. This has led to a broad clinical spectrum ranging from mild upper respiratory tract infections to fatal systemic inflammation [7,8]: HCoV-229E, NL63, OC43, and HKU1 have long circulated in temperate regions during winter and spring, causing only common colds in adults or bronchiolitis in infants, but due to their short-lived immune memory, they maintain a high reinfection rate, continuously consuming public health resources [9,10]. In contrast, highly pathogenic beta-coronaviruses such as SARS-CoV, MERS-CoV, and SARS-CoV-2 have acquired efficient human-to-human transmission capabilities through adaptive mutations in the RBD and furin cleavage sites, triggering systemic inflammatory response syndrome centered on acute lung injury, cytokine storms, and thrombotic microangiopathy [11], with a fatality rate of 3–35%, and survivors suffer long-term disease burdens such as pulmonary fibrosis, cardiometabolic disorders, and neurocognitive deficits [12]. Their large-scale epidemics not only directly increase excess mortality but also cause profound socio-economic trauma through labor force loss, healthcare system strain, and trade disruptions [13]. In the animal domain, alpha-coronaviruses such as porcine epidemic diarrhea virus (PEDV) and transmissible gastroenteritis virus (TGEV), and delta-coronavirus PDCoV, exhibit nearly 100% lethality in newborn piglets. After invading through the intestinal epithelial ACE2 pathway, they rapidly destroy the villus–crypt structure, causing osmotic diarrhea, dehydration, and metabolic acidosis [14], A single outbreak can wipe out an entire batch of suckling piglets in large-scale pig farms, causing a sudden and devastating loss across the production chain in the industrial chain. Avian gamma-coronavirus infectious bronchitis virus, through recombination and mutation, has continuously broken through tissue tropism restrictions, expanding from the classic respiratory and renal types to reproductive and proventricular types [15], leading to a sudden drop in egg production in laying hens, restricted weight gain in broilers, and carcass rejection, causing an average annual economic loss of over one billion US dollars in the global poultry industry. More alarmingly, in scenarios of intensive farming and wildlife trade, coronaviruses continue to undergo genetic recombination and point mutations. Canine coronavirus and porcine delta-coronavirus porcine delta coronavirus (PDCoV) have been detected in patients with pneumonia in Malaysia and febrile children in Haiti [16,17], respectively, with a high similarity rate of genomic to human strains, suggesting their potential for direct cross-species infection. Thus, coronaviruses not only pose a threat to individual health ranging from mild rhinitis to multi-organ failure but also, due to their cross-species transmission ability and genomic plasticity, represent a long-term risk source for livestock biosecurity and the global public health system.

The replication process of coronavirus is complex. The virus releases RNA through membrane fusion, and then synthesizes RNA polymerase and replicates the viral genome, encoding non-structural proteins (NSPs) and structural proteins. The last third of the coronavirus genome mainly encodes four structural proteins: spike (S) protein, envelope (E) protein, membrane (M) protein and nucleocapsid (N) [18], They play roles in invasion, assembly, envelope formation and RNA stability [19]. In addition, the pathogenic mechanism of coronaviruses lies more in the overheated immune response it triggers. The genome and proteins of the virus are recognized by the pattern recognition receptors (PRRs) of the host cell, and the host induces the expression of type I interferons (IFN-I), pro-inflammatory factors and antiviral genes to inhibit viral replication by regulating immune signaling pathways. However, coronaviruses have developed complex immune escape mechanisms that evade immune clearance by encoding proteins that antagonize or delay the host antiviral interferon system, which is key to their high lethality [20].

The host’s innate immune system is the first line of defense within hours of pathogen invasion, inhibits early viral replication and spread, and regulates the initiation and development of acquired immunity. Innate immunity is essential for infection control during the 4–7-day interval between the initial stage of infection and the onset of acquired immunity. The immune response mechanisms triggered by SARS-CoV-2, such as cytokine storms, abnormal neutrophils and lymphocyte imbalances [11,21], perfectly demonstrate the “multi-node, multi-pathway” escape strategy utilized by coronaviruses. They can even inversely regulate the innate immunity of the host, thereby laying the pathological foundation for its wide spread and high rate of severe cases [22]. However, these mechanisms are not unique to SARS-CoV-2: from SARS-CoV, MERS-CoV to the novel coronaviruses that have been continuously recombining in the animal reservoirs recently, similar immune escape strategies have been reproduced to varying degrees [23,24]. Therefore, here we comprehensively review the innate antiviral immune response of the body, as well as the escape and antagonistic suppression of the innate immune system by SARS-CoV-2-dominated coronaviruses, so as to systematically and comprehensively reveal the infection and immune escape mechanism of coronaviruses. This review aims to extend the experience gained during the COVID-19 pandemic into a “general blueprint for immune evasion of coronaviruses”, to describe the ongoing evolutionary game between the virus and the host from a more comprehensive perspective, with the expectation of providing a more scientific basis for future prevention, control and treatment.

## 2. Host Innate Antiviral Immune Responses

The innate immune system is the host’s first line of defense against pathogens, providing immediate non-specific defense. The main components of the innate immune system include physical barriers (such as skin and mucous membranes), innate immune cells (such as macrophages, dendritic cells, neutrophils, and natural killer cells), and a series of molecular PRRs and antimicrobial molecules (such as complement molecules, antimicrobial peptides and acute phase proteins). When the virus invades the host, the innate immune system recognizes pathogen-associated molecular patterns through different types of PRRs. This is followed by a cascade of reactions that promote the expression of interferons (IFNs) and pro-inflammatory cytokines to fight viral infection and coordinate adaptive immunity, thereby limiting the spread of the virus.

### 2.1. PRRs

**Figure 1:**
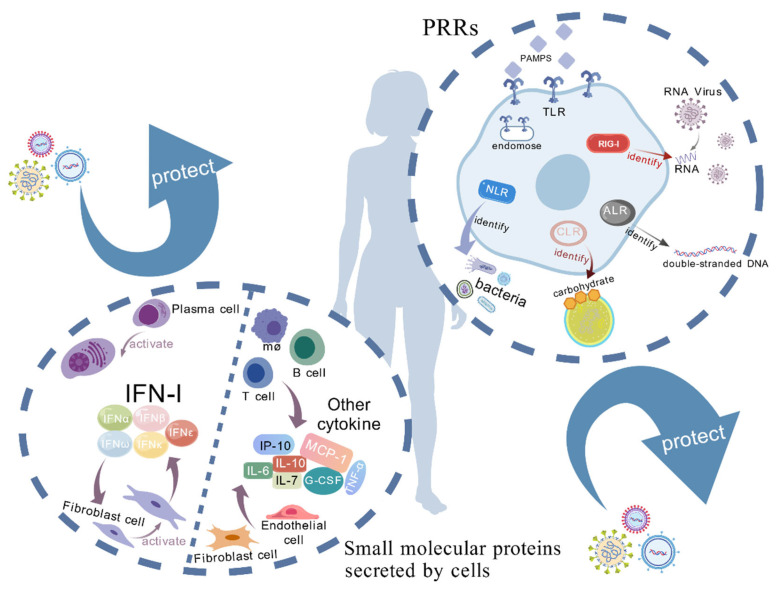
PRRs and small molecular proteins secreted by cells. (i) PRRs: Host cells contain various pattern recognition receptors (PRRs); Toll-like receptors (TLRs) are located on the cell membrane and endosomal membranes, recognizing different viral PAMPs to induce the production of IFN-β and inflammatory cytokines, thereby restricting viral replication. RIG-I recognizes viral RNA in the cytoplasm, activating the IRF3 and NF-κB pathways to promote the production of type I interferons. NOD-like receptors (NLRs) recognize intracellular viral components and danger signals, participating in inflammasome formation. C-type lectin receptors (CLRs) recognize glycosylated pathogen components, affecting viral uptake and processing. AIM2 recognizes intracellular double-stranded DNA, activating the inflammasome to produce IL-1β and IL-18. These PRRs collectively constitute the antiviral defense system of host cells. (ii) Small molecular proteins secreted by cells: Host immune cells (such as macrophages, dendritic cells, B cells, T cells, etc.) as well as epithelial cells and fibroblasts produce type I interferons (IFN-I, including IFN-α and IFN-β) and cytokines (such as TNF-α, IL-6, etc.) upon viral infection. These molecules can directly inhibit viral replication, enhance inflammatory responses, attract immune cells to the site of infection, and promote viral clearance. This figure was created by BioGDP.com (https://biogdp.com/workspace, accessed on 22 November 2025) [36].

PRRs in the innate immune system can specifically recognize PAMPs, such as lipopolysaccharides, peptidoglycans, lipoproteins, and flagellins of bacteria, as well as double-stranded RNA (dsRNA) and unmethylated CpG DNA of viruses. Activation of PRRs triggers a series of signaling events that ultimately lead to the initiation of the host innate immune response, including the production of inflammatory factors and activation of immune cells [25]. PRRs include Toll-like receptors (TLRs), RIG-I-like receptors (RLRs), NOD-like receptors (NLRs), C-type lectin receptors (CLRs), AIM2-like receptors (ALRs), and GMP–AMP synthase (cGAS)-like receptors (cGLRs) (Table 1). They form a highly coordinated and finely regulated network in the pathogen monitoring and host defense mechanism, jointly ensuring effective host recognition and rapid response to pathogens [26,27] (Figure 1).

Previous studies have confirmed that in the early stage of viral infection, PRRs such as TLR3, TLR7, RIG-I, and melanoma differentiation-associated gene 5 do not act independently but rather initiate the antiviral response through collaborative cascades [28]. For instance, when an RNA virus invades, RIG-I first recognizes short-chain RNAs with a triphosphate terminus and rapidly transmits activation signals through the mitochondrial antiviral signaling (MAVS) pathway [29,30]; at the same time, it induces TLR7 to be transported to the endosomal membrane. After the virus is internalized, this further validates the pathogen, and the dual pathways synergistically amplify the signal, prompting IFN-I to rapidly reach high concentrations, directly inhibiting viral replication [31]. Moreover, the function of PRRs is dynamically regulated by post-translational modifications: when lactic acid accumulates, the AARS1/2 enzymes can temporarily inhibit the DNA sensing ability of cyclic cGAS through lacotylation modification of specific lysine residues of cGAS [32], while when the intensity of viral infection is high, the ubiquitin-conjugating enzyme E2 M-mediated ubiquitination (neddylation) modification of RIG-I can enhance its stability and signal persistence, and this “fast-slow regulation” mechanism ensures that IFN-I takes effect promptly and avoids long-term damage to host tissues [33].

On the other hand, the previously overlooked inhibitory PRRs play a crucial role in the regulation of immune homeostasis [34,35]: receptors such as Siglec-10 and CEACAM1, which contain immunoreceptor tyrosine inhibitory motifs, when bound to viral glycoproteins or host damage-related molecules, can recruit SHP-1/2 phosphatases, directly antagonizing the activation signals of the TLR and CLR pathways, and preventing the occurrence of cytokine storms. This indicates that the host can amplify the antiviral response by “lifting immune suppression” rather than relying solely on activating pathways. In summary, the PRR network is like an “immune system” with dynamic regulatory functions: the effector pathways centered on activated receptors, and the inhibitory receptors combined with post-translational modifications constitute a balanced regulatory mechanism. Only when all components cooperate can an efficient and safe antiviral immune response be achieved.

### 2.2. Small Molecular Proteins Secreted by Cells

Both IFNs and other cytokines, which play a central role in host innate immunity, are small molecular proteins secreted by cells and play important roles in the immune system. Among them, IFN-I (such as IFN-α and IFN-β) is rapidly produced by the host cell after recognizing viral RNA or DNA. These IFNs activate signal transduction pathways by binding to receptors on the cell surface, inducing the expression of Interferon-Stimulated Genes (ISGs), and the proteins encoded by these genes can directly inhibit viral replication or regulate the function of immune cells, thus limiting the spread of viruses in the host [37]. In antiviral immunity, other cytokines (such as tumor necrosis factor α (TNF-α), interleukin (IL)) can enhance the inflammatory response, attract immune cells to the site of infection, promote virus clearance, and participate in the activation of adaptive immunity [38]. There is a complex cross-regulatory relationship between IFN and other cytokines. IFN not only directly inhibits the virus, but also regulates the inflammatory response by influencing the production of other cytokines. For example, IFN can induce the production of certain cytokines that further activate immune cells and enhance the antiviral response [38,39]. At the same time, certain cytokines can also affect the activity of the IFN signaling pathway, forming a feedback loop to maintain the appropriate intensity and duration of the immune response [40] (Figure 1).

During viral infection, the balance of IFN and other cytokines is critical to effectively clear the virus and avoid immunopathology. For example, in SARS-CoV-2 infection, insufficient early production of IFN may lead to uncontrolled viral replication, while a “cytokine storm” late in infection may lead to severe immune damage [39,40]. Therefore, understanding the role and regulatory mechanism of IFN and other cytokines in innate antiviral immunity is of great significance for developing new antiviral therapeutic strategies. It is worth noting that Wei et al. utilized single-cell multi-omics combined with CRISPR screening technology to identify a group of “super-inducing” ISGs in the bronchoalveolar lavage fluid of critically ill COVID-19 patients, such as new alternative splicing variants of *IFI44L* and *IFITM3* [41]; these ISGs not only have broad-spectrum anti-coronavirus activity themselves, but also can enter adjacent uninfected cells in the form of exosomes to form a lateral protection network, further providing a new target for intervention in the COVID-19 infection model.

### 2.3. MicroRNAs (miRNAs)

**Figure 2:**
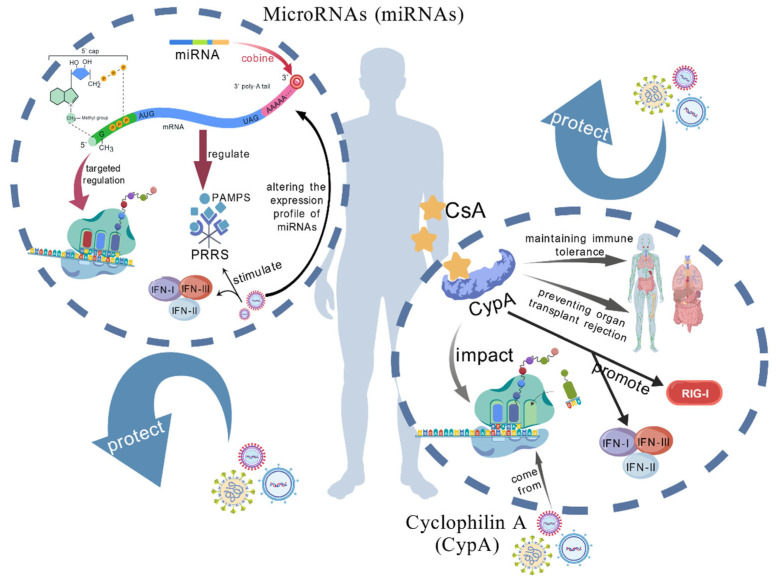
MicroRNAs and Cyclophilin A. (i) MicroRNAs (miRNAs): miRNAs regulate gene expression by binding to the 3′-UTR of target mRNAs. They function as part of the host defense mechanism against viral infections but can also be exploited by viruses to promote their life cycle. miRNAs can directly target viral RNA or proteins to modulate viral replication, transcription, and translation. They also regulate host antiviral signaling pathways to maintain immune homeostasis. Additionally, miRNAs can influence viral entry, replication, assembly, and release. Viral infections can significantly alter the expression profiles of miRNAs in host cells, changes that are closely related to host antiviral immunity and the viral life cycle. (ii) Cyclophilin A (CypA): Cyclophilin A (CypA)in the host serves as the intracellular receptor for cyclosporin A (CsA), maintaining immune tolerance and preventing organ transplant rejection. It also directly participates in the viral replication process and modulates the host’s innate immune response. During influenza virus infection, CypA regulates the RIG-I and MAVS signaling pathways to further promote the production of IFN-I. This figure was created by BioGDP.com [36].

miRNAs, as a class of small non-coding RNA, regulate gene expression by binding to the 3′-untranslated region (3′-UTR) of the target mRNA, thereby affecting the function of cells. In the context of viral infection, miRNAs not only act as a kind of host defense mechanism, but may also be utilized by the virus to facilitate its life cycle [42]. miRNAs participate in the whole process of antiviral innate immune response through various regulatory mechanisms, including direct targeting of viral molecules, regulation of host antiviral signaling pathways, and adjustment of host cell immune status [43,44,45] (Figure 2).

Studies have shown that these molecules are not only key defense carriers of the host’s innate immunity, but they may also be hijacked by coronaviruses to accelerate the infection process: Host miRNAs can resist infection by precisely targeting the viral genome or regulating immune signaling pathways. For example, after infection with the SARS-CoV-2 Delta variant strain, the expression of *miR-155-5p* and others increases, which enhances the type I interferon effect by regulating the NF-κB pathway and *IFIT1/2* and other antiviral genes, and their expression differences are closely related to the pathogenicity of the virus; coronaviruses can also achieve immune evasion through their own encoded miRNAs or by utilizing the host miRNA network. For instance, the viral-derived small RNA *miR-nsp3-3p* encoded by SARS-CoV-2 can exacerbate pulmonary fibrosis by inhibiting Activated Leukocyte Cell Adhesion Molecule expression in severe patients [46]. In the face of this dual effect similar to an “immune regulation knob”, how to strike a balance in their expression is the key point of future research.

### 2.4. Cyclophilin A (CypA)

CypA is a widely existing protein with peptidyl prolyl cis-trans isomerase activity, which is involved in protein folding and signal transduction [47]. As an intracellular receptor of immunosuppressant Cyclosporine A (CsA), CypA plays an important role in maintaining immune tolerance and preventing organ transplantation rejection [48]. In addition, CypA is also involved in biological processes such as viral infection, inflammation and tumorigenesis [49].

CypA plays multiple roles in the host’s antiviral response. Studies have shown that CypA can interact with proteins of various viruses and affect the process of viral replication and infection [48,50]. For example, CypA interacts with NSPs of the hepatitis C virus (HCV) and is a host factor necessary for HCV replication. In coronaviruses, CypA has also been confirmed to be crucial for the replication of SARS-CoV, CoV-229E, CoV-NL63, feline coronavirus and other coronaviruses [51]. In addition, CypA not only directly participates in viral replication, but also influences viral infection by regulating the host’s natural immune response [52]. Studies have found that CypA can promote the RIG-I mediated antiviral innate immune signaling pathway, and promote IFN-I production by enhancing K63 ubiquitination and the stability of RIG-I, thus enhancing host defense against viruses [49].

It is worth noting that the role of CypA in various types of virus infection is different [49]. In influenza virus infection, CypA promotes IFN-I production by regulating the RIG-I and MAVS signaling pathways. In addition, CypA can also interact with Group A streptococcus (GAS) to promote bacterial adhesion and invasion, indicating that CypA also plays a role in virus-induced bacterial co-infection. CypA is a potential target for antiviral therapy, and its inhibitors such as CsA and its derivatives have been shown to have significant inhibitory effects on a variety of coronaviruses [53,54]. This suggests that by targeting CypA, new antiviral drugs may be developed. Lydia et al. demonstrated that PROTACs (CG167 and RJS308) can selectively degrade host CypA by recruiting the VHL E3 ubiquitin ligase. In HIV-1 and HCV infection models, these PROTACs exhibited superior antiviral activity compared to traditional inhibitors. Additionally, the compounds feature simple synthesis and reduced immunosuppressive side effects [55]. However, the versatility and widespread expression of CypA in host cells also present challenges that require careful consideration of therapeutic strategies to avoid adverse effects on normal host function (Figure 2).

## 3. The Escape Strategies of Coronavirus Pertaining to Innate Immunity

After understanding how the host’s innate immunity quickly sets up defenses against the invasion of viruses through the steps of “recognizing the virus–signal transmission–triggering the response”, the question naturally turns to how the virus “counteracts each defensive measure”. The escape mechanism of coronavirus to the innate immune system can be divided into two types: escape and antagonism. In the long struggle with the host, coronaviruses have evolved multiple escape strategies through which they are able to replicate and spread efficiently within the host body while reducing the effective response of the host immune system.

### 3.1. The Escape Mechanisms of Coronavirus to the Innate Immune System

Coronaviruses employ coordinated mechanisms to evade host innate immunity: evasion of immune surveillance, suppression of MAVS functionality, disruption of PRR activation, viral evolution through mutation, and so on. These synergistic strategies—by impairing antigen presentation, hijacking immune signaling cascades, and subverting molecular pattern recognition—create immunological blind spots that facilitate viral immune escape.

After experiencing the COVID-19 pandemic, researchers have gained a deeper understanding of the adaptability of coronaviruses. They constantly switch between offense and defense in their interactions with the host, as exemplified by their hijacking of miRNA and CypA mentioned earlier [56]: miRNA can fine-tune antiviral genes to help the host inhibit viral infection, while it may also be hijacked by the virus to shut down the interferon pathway; in contrast, CypA, which normally aids in immune activation, can be exploited by the virus to encapsulate itself and evade immune recognition. From such a perspective, coronaviruses have demonstrated a highly conserved and continuously refined immune evasion strategy. Crucially, the evolutionary tactics driving these adaptations illuminate actionable targets for next-generation vaccine design and precision antiviral therapies.

#### 3.1.1. Evasion of Immune Surveillance

##### The Decreased Expression of Major Histocompatibility Complex Class I (MHC-I) Molecule

MHC-I molecules exist on the cell surface and their main function is to present peptides produced within the cell to CD8^+^ T cells. These peptides are usually derived from the cell’s own proteins, but can also include proteins from viruses or other pathogens [57]. In this way, the MHC-I molecule helps the immune system recognize and destroy infected or abnormal cells. The MHC-I molecule consists of a heavy chain containing two polypeptide-binding domains and an immunoglobulin-like domain, and a light-chain β2-microglobulin. Human MHC-I molecules are typically encoded by the *HLA-A*, *HLA-B*, and *HLA-C* genes, which are highly polymorphic, i.e., there are many different variants, which increase the ability to recognize different antigens between individuals [58].

**Figure 3:**
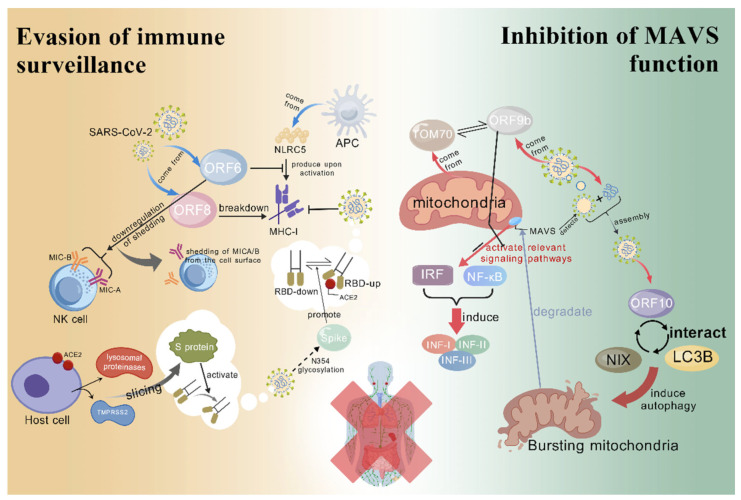
Evasion of immune surveillance and inhibition of MAVS function. (i) Evasion of immune surveillance: The virus uses its ORF6 protein to inhibit the function of NLRC5 and downregulate the NKG2D ligands MIC-A/B, thereby reducing the expression of MHC-I molecules in the host and evading NK cell surveillance, thus minimizing the killing of infected cells. Additionally, the virus’s ORF8 protein interferes with signaling through autophagy, further weakening the host immune response. Moreover, the virus can hide neutralizing epitopes through conformational changes in the RBD, reducing antibody binding efficiency and evading humoral immunity. Some viral proteins also rely on host proteases for activation, thereby regulating viral entry, replication, and assembly processes to ensure efficient proliferation and transmission within host cells. (ii) Inhibition of MAVS function: MAVS, located on the outer mitochondrial membrane, can sense the replication intermediates of RNA viruses and activate downstream IRF and NF-κB signaling pathways, thereby inducing the production of type I interferons and other antiviral factors. The ORF9b of SARS-CoV-2 can interact with the mitochondrial protein TOM70 to inhibit downstream MAVS signaling and block the production of type I interferons. ORF10, on the other hand, induces mitophagy by interacting with NIX and LC3B, degrading MAVS, and thereby inhibiting innate immune signaling. This figure was created by BioGDP.com [36].

During viral infection, the expression of MHC-I molecules is critical for the activation of cytotoxic T cells. Virus-infected cells display viral antigen peptides on the cell surface via MHC-I molecules, triggering an immune response [59]. As a result, coronaviruses reduce the expression of MHC-I molecules through a variety of mechanisms to evade the surveillance of the immune system. These mechanisms include the following: (i) The role of ORF6 protein: The ORF6 protein encoded by SARS-CoV-2 can inhibit the function of NLRC5 protein in host cells, and then affect the expression of MHC-I molecules. NLRC5 is a key transcription factor that activates the MHC-I pathway, and its inhibition reduces the expression of MHC-I molecules, helping the virus evade cytotoxic T cell recognition [60]. (ii) The effect of ORF8 protein: The ORF8 protein of SARS-CoV-2 locks MHC-I molecules into lysosomal degradation through autophagy, which reduces the sensitivity of virus-infected cells to cytotoxic T lymphocytes. This mechanism is directly involved in the degradation of viral RNA and reduces the presentation of viral antigens. [61] (iii) Interference with signal transduction pathways: coronavirus may also reduce the expression of MHC-I molecules by interfering with host signal transduction pathways, which involve the regulation of host transcription factors by viral proteins, and may affect host transcription factors by other viral proteins, thus affecting the transcription of MHC-I-related genes (Table 2) [60,61]. These mechanisms work together to enable coronavirus to replicate in the host and reduce the likelihood of it being cleared by the host immune system. Therefore, studying these escape mechanisms is of great significance for the development of new therapeutic strategies and vaccines (Figure 3).

##### Immune Stealth: Dynamic Changes In RBD

In addition to evading immune surveillance by regulating host antigen-presenting molecules, coronaviruses can also “disguise” themselves by altering the conformation of their own proteins. The most representative example of this is the dynamic change in the RBD region. RBD is part of the S protein on the surface of the virus and is responsible for binding to the ACE2 receptor on the surface of the host cell, thereby mediating the entry of virus into cells.

RBD can switch between the “up” and “down” states, and this dynamic change in conformation enables SARS-CoV-2 to achieve the effect of “immune stealth”, thus escaping the surveillance of the host innate immune system [3]. In the “up” state, RBD is exposed to the surface of the virus and can effectively bind to the ACE2 receptor, thus promoting the virus to invade the host cell. In the “down” state, RBD is hidden, reducing the chance of being recognized and neutralized by the host immune system. This dynamic change in the conformation of RBD helps spread the virus through the host while reducing the risk of it being cleared by the innate immune system [62].

It is worth noting that SARS-CoV-2 significantly enhanced the adaptability of the virus to humans by increasing glycosylation modification, especially N354 glycosylation on the S protein [63]. N354 glycosylation reduces viral infectivity by changing the conformational state of RBD, thereby adjusting the adaptability of the virus in the host. The above-mentioned glycosylation modification further masks key antigen epitopes, which not only improves the immune escape ability of the virus, but more importantly, reduces the immunogenicity of the virus, thus further weakening the immunoblotting of the host, and ultimately enables the virus to re-infect in the future (Table 2). In addition, N-linked glycan obtained on the RBD of SARS-CoV-2 is a novel means of immune escape and should be closely monitored during viral antigen drift [64].

##### Utilization of Host Proteases

SARS-CoV-2 can also use proteases within the host cell to activate the S protein on its surface (Table 2). Indeed, host proteases such as TMPRSS2 and lysosomal proteases are able to cut the S protein on the surface of the virus, a process that is critical for the virus to enter the host cell. The S protein of SARS-CoV-2 is activated by the cleavage of the host protease, exposing RBD, which can efficiently bind to the ACE2 receptor on the surface of the host cell and promote viral invasion [3].

##### Evasion of Natural Killer Group 2D (NKG2D)-Mediated Cytotoxic Immunity

Furthermore, for the “relatives” of the novel coronavirus—other coronaviruses belonging to the β-coronavirus lineage B (such as the SARS virus), there are also similar ways to evade the immune surveillance of the body through host proteins: for example, the utilization of NKG2D. NKG2D is an activating receptor expressed on NK cells and certain T cells that recognizes and binds to stress-inducing ligands on the cell surface, such as MIC-A and MIC-B, to trigger cytotoxic responses. And this response is essential for clearing virus-infected cells.

It has been found that SARS-CoV-2 and other beta coronaviruses evade NKG2D-mediated cytotoxic immunity by down-regulating the NKG2D ligands MIC-A and MIC-B on the cell surface (Table 2) [65]. This downregulation occurs through proteolytic shedding, resulting in MIC-A and MIC-B shedding from the surface of infected cells, thereby reducing the activation of the NKG2D receptor. It has also been suggested that ORF6, an accessory protein of SARS-CoV-2, plays a key role in this escape mechanism [65]. ORF6, the only conserved helper protein in the beta group of coronaviruses, is responsible for the downregulation of MIC-A and MIC-B through proteolytic cleavage. Although the virus uses this mechanism to escape immune surveillance, NK cells are still able to effectively recognize and kill SARS-CoV-2-infected cells, limiting the spread of the virus. In addition, inhibition of MIC-A and MIC-B shedding using the monoclonal antibody 7C6 can further enhance the activity of NK cells against virus-infected cells.

These findings reveal strategies by which beta coronaviruses evade NKG2D-mediated cytotoxic immunity and identify ORF6 as a shared immune escape protein, which is helpful for understanding how the virus adapts to the host immune system and may guide the development of new antiviral treatment strategies.

#### 3.1.2. Inhibition of MAVS Function

MAVS are key signaling molecules that activate host innate immunity during RNA virus infection. Located in the outer mitochondrial membrane, MAVS can sense RNA replication intermediates of RNA viruses and activate downstream interferon regulatory factor (IRF) and NF-κB signaling pathways, thereby inducing the production of IFN-I and other antiviral factors [66].

Coronaviruses inhibit the function of MAVS through different strategies, including directly interacting with MAVS, blocking their binding to downstream signaling molecules, or degrading MAVS by inducing mitochondrial autophagy [67]. For example, NSP ORF9b of SARS-CoV-2 is able to interact with the mitochondrial protein translocase of the outer mitochondrial membrane 70 (TOM70) to inhibit MAVS downstream signaling, thereby inhibiting IFN-I production. In addition, the ORF10 protein of SARS-CoV-2 induces mitochondrial autophagy by interacting with NIP3-like protein X and Microtubule-associated protein 1 light chain 3 beta, thereby degrading the expression of MAVS, blocking the innate immune signal transduction, and inhibiting the innate immune response to further inhibit IFN-I signal response [68]. Summarily, these mechanisms help the coronavirus replicate within host cells while reducing the host’s antiviral response, thereby evading surveillance and clearance by the innate immune system. By inhibiting MAVS, the coronavirus is able to reduce the production of IFNs in the early stages of infection, creating favorable conditions for the spread of the virus and the development of the disease (Figure 3).

#### 3.1.3. Interferes with PRR Activation

During the replication of coronavirus, the dsRNA intermediates generated activate PRRs and trigger the innate immune response of neighboring cells. To circumvent innate immune responses, coronaviruses must adopt strategies to avoid being recognized by the PRRs. It is revealed that several coronaviruses, including SARS-CoV and mouse hepatitis virus, are able to replicate within small vesicles with a bilayer membrane structure, a mechanism that may be designed to hide virus-associated PAMPs from detection by PRRS in the cytoplasm.

**Figure 4:**
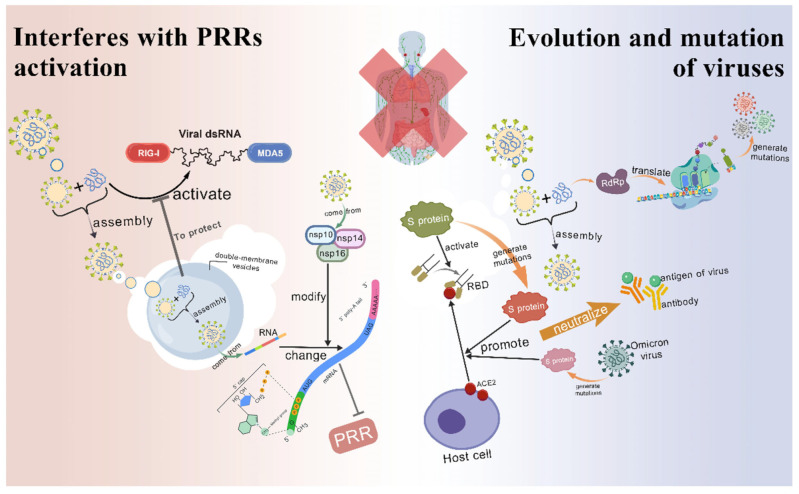
Interferes with PRR activation and evolution and mutation of viruses. (i) Interferes with PRR activation: To evade the activation of PRRs by double-stranded RNA intermediates produced during replication, coronaviruses replicate within double-membrane vesicles to hide virus-related PAMPs. Additionally, coronaviruses mimic the host’s capping mechanism, using the nonstructural proteins nsp14 and the nsp16/nsp10 complex to modify the RNA cap structure, thereby enhancing the stealthiness of viral RNA and effectively evading the host’s innate immune response. (ii) Evolution and mutation of viruses: Due to the weak proofreading ability of RdRp and high recombination frequency, coronaviruses exhibit numerous genetic mutations, resulting in diverse progeny. Some variants evade host immune surveillance by altering the antigenicity of the surface S protein. Moreover, the spike protein mutations in the Omicron variant enhance its binding capacity to the host cell receptor ACE2, thereby increasing infection efficiency. This figure was created by BioGDP.com [36].

One of the characteristics of eukaryotic mRNA is the N7-guanosine methylated cap structure (CAP), including CAP-0 and CAP-1, which is not only a marker of RNA polymerase II transcripts, but also an important basis for host cells to distinguish their own RNA from foreign RNA. In order to evade the recognition of the host PRRs, many viruses mimic the host capping mechanism and modify their own RNA. In vitro experiments have confirmed that the NSP14 and NSP16/NSP10 complex of SARS-CoV play a key role in the RNA capping process [69] (Figure 4).

The synthesis of RNA cap structures involves a series of enzymatic reactions, including the synergistic action of RNA triphosphatase, guanosine transferase, and N-7 guanosine methyltransferase. During the capping process of SARS-CoV, the NSP14 protein acts as an N7-guanosine methyltransferase, which is responsible for methylating the N-7 position of the RNA cap structure to form a CAP-0 structure highly similar to the host RNA cap, which makes it difficult for the host to distinguish viral RNA from its own RNA [70]. In addition, NSP16 protein also possesses 2′-O-methyltransferase activity, which can further modify the cap structure of viral RNA and enhance the concealability of viral RNA, thus effectively escaping the host’s innate immune response [71,72].

#### 3.1.4. Evolution and Mutation of Viruses

As a class of enveloped RNA viruses, coronaviruses have shown remarkable evolutionary and mutational capabilities, which to a large extent is a key strategy for them to adapt to the pressure of the host innate immune system and evade immune surveillance [73]. In particular, coronaviruses that can spread across species and cause disease in humans, such as SARS-CoV, MERS-CoV, and SARS-CoV-2, have evolved multiple mechanisms to ensure their survival and spread (Figure 4).

##### The Rapid Evolution of RNA Viruses

During the replication of SARS-CoV-2, the mutation rate of SARS-CoV-2 virus was reduced due to the strong and relatively stable proofreading ability of NSP14 [74]. Despite this correction mechanism, the ability of RNA-dependent RNA polymerase (RdRP) to proofread and fix errors during replication is relatively weak. In addition, RNA viruses have a high recombination frequency, which leads to a significant increase in genetic diversity and is prone to replication errors when the virus replicates [75]. These errors often lead to mutations in the gene sequence, and the high replication rate of RNA viruses further increases the likelihood of mutations. The high mutation rate allows coronaviruses to rapidly produce diverse offspring, some of which may gain the ability to evade the surveillance of the host immune system, for example, by altering the antigenicity of their surface proteins, thereby reducing the effectiveness of vaccines and therapies, making them less easily recognized and cleared [76].

##### Variation in S Protein

The S protein of coronavirus is an important structural protein on the surface of the virus, responsible for recognizing receptors on the surface of the host cell and mediating the binding of the virus to the host cell. Mutations in the S protein can affect a virus’s transmissibility, pathogenicity, and ability to evade the host’s immune system.

It has been shown that variant strains of the novel coronavirus (SARS-CoV-2), such as Delta (B.1.617.2) and Omicron (B.1.1.529), enhance the binding ability of the host cell receptor ACE2 through mutations in the S protein, which not only improves the efficiency of the virus infection, but may also lead to an expansion of the host range of the virus for different species [77]. In addition, these mutations may also help the virus evade neutralization by neutralizing antibodies, thereby enhancing immune escape [78]. For example, Omicron mutant strains showed a large number of mutations in the S protein, which enhanced binding to human ACE2, and showed varying degrees of resistance to neutralizing antibodies in the serum of recovered patients, showing characteristics of immune escape [79].

The transmission ability of these variant strains has increased, partly due to the enhanced antagonistic effect of their molecular-level interaction with the host’s innate immune response. This is more evident in the recently mutated JN.1, BA.2.86, and other virus strains. The immune evasion characteristic of the JN.1 variant mainly lies in precise evasion of T-cell immunity. It significantly weakens the host’s immune recognition through the complex mutation of key immune epitopes. This variant strain carries a composite mutation of N450D/L452W/L455S in the immunodominant region of the S protein, HLA-A24-S_448–456_. By altering the hydrophobicity of the peptide and interfering with the binding and recognition of the T-cell receptor (TCR), it induces significant T-cell immune evasion, especially reducing the T-cell immune response in the HLA-A24 population. Simultaneously, a Q229K mutation occurred for the first time at the highly conserved Q229 site on the N protein. This mutation is located in the TCR recognition region and further affects the host’s subsequent targeted immune response. These dual mutations jointly strengthen its immune evasion ability [80]. The BA.2.86 variant also possesses significant immune evasion characteristics and shares evolutionary associations and differences with JN.1. As the precursor variant of JN.1, it carries the N450D/L452W mutation in the same immunogenic region of the S protein, but this mutation does not significantly affect the recognition of the viral peptide by the T-cell receptor and still retains most cross-immune responses [80]. However, both BA.2.86 and JN.1 carry the Q229K mutation on the N protein. This mutation disrupts the conserved T-cell immune epitope in the Sarbecovirus subgenus, thereby affecting the host’s immune recognition of the virus. Moreover, BA.2.86 has been confirmed to differentiate into a new serotype (VI type), and its antigenic profile is significantly different from previous strains, indicating that vaccines based on the XBB antigen have an escape risk for it and its subsequent derivative strains [81].

In summary, the mutation of coronavirus S protein has an important impact on the spread and pathogenicity of the virus by enhancing the ability of the virus to bind to the host cell and evade the host immune surveillance. These variants pose challenges to the effectiveness of vaccines and treatment strategies, underscoring the importance of continuous monitoring of virus variants and updating public health measures.

### 3.2. Antagonistic Mechanisms of Coronavirus on Innate Immune Responses

**Figure 5:**
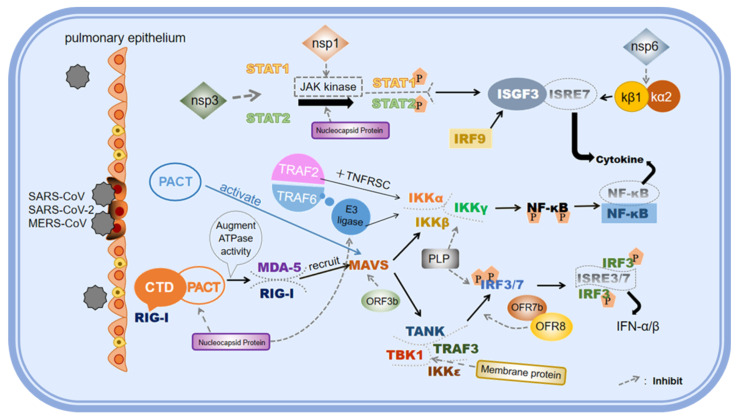
The antagonistic mechanisms of coronavirus on innate immune responses. Coronaviruses (such as SARS-CoV, SARS-CoV-2 and MERS-CoV) deeply intervene in the host cell signaling mechanisms through their structural proteins, NSPs and other accessory proteins, thereby achieving immune evasion strategies. This figure was created by BioGDP.com [36].

Based on immune evasion, many exogenous microorganisms have further evolved mechanisms that counteract the host’s innate immune response. This is also one of the key strategies for their survival and dissemination. It has been revealed that the antagonistic mechanism of coronaviruses is mainly through their self-encoded proteins, using a variety of direct and indirect mechanisms to effectively inhibit the generation of IFNs and the immune signaling pathway regulated by IFNs (Figure 5). Clinical observation shows that the level of IFN-I in patients infected with coronavirus is significantly reduced, especially in severe patients infected with SARS-CoV-2, SARS-CoV and MERS-CoV [82]. In vitro experiments have further confirmed that SARS-CoV and MERS-CoV can inhibit the expression of IFN-I in host cells to varying degrees, while the replication of coronavirus can be effectively inhibited by exogenous addition of IFN-α [83]. These findings are supported by clinical studies showing that IFN-α in combination with other antiviral drugs can significantly improve the survival rate of patients with coronavirus infection, which strongly supports the crucial role of IFN-I in the resistance to coronavirus infection [84].

After the coronavirus infects the host cell, its genome is released and used to replicate and express related proteins, while acting as PAMPs recognized by PRRs [85]. PRRs such as TLR3, TLR7, TLR8, TLR9, RIG-I and MDA5 in host cells bind to the coronavirus genome and transmit antiviral signals through their respective adaptor proteins [39,86]. Of course, not all viral proteins of coronaviruses are involved in immune suppression. For instance, many proteins of SARS-CoV-2, such as NSP1, NSP6, and NSP8, play a significant role in immune evasion. However, the detailed mechanisms of immune evasion for NSP5, ORF3b, and ORF9c have not been fully elucidated, suggesting that these proteins may not have as clear an immune evasion function as other known immune-inhibiting proteins [87].

The emergence of SARS-CoV has aroused global attention of coronaviruses, and the studies of MERS-CoV and SARS-CoV-2 have further deepened the understanding of the relationship between coronavirus and innate immunity. Within host cells, coronaviruses not only inhibit the production of IFN-I, but also prevent the degradation of host RNA by inhibiting the nonsense-mediated mRNA decay (NMD) pathway [88], which is a way of intracellular antiviral activity. In addition, coronaviruses antagonize the innate immune response of the host through a variety of mechanisms, including direct or indirect inhibition of IFNs and their mediated immune signaling pathways [89,90], which are essential for the survival and spread of the virus.

#### 3.2.1. Antagonism Mediated by Structural Proteins

The structural proteins of the coronavirus family, especially the M protein and N protein, play a crucial role in many aspects of the viral life cycle. These proteins not only participate in the assembly and structural maintenance of virus particles, but also achieve effective avoidance of the host innate immune system by finely regulating the host immune response. M and N proteins significantly inhibit the production and signal transduction of IFNs by directly acting on key molecules of host immune signaling pathways [91], a process that involves a variety of complex molecular mechanisms.

SARS-CoV-2’s M protein is able to inhibit the production of type I and III IFNs by targeting intracytoplasmic RIG-I/MDA5-mediated RNA virus recognition pathways, thereby enabling immune escape. By directly interacting with signal transduction molecules such as RIG-I, MAVS, TANK-binding kinase 1 (TBK1), and tumor necrosis factor receptor-associated factor 3 (TRAF3), the M protein effectively prevents the formation of the RIG-I/MAVS/TRAF3/TBK1 complex, thereby inhibiting the phosphorylation and nuclear translocation of IRF3. In addition, transcriptional activation of type I and type III IFN-inducing genes was also inhibited [92]. This mechanism reveals the key role of M protein in regulating IFN signaling pathway, thereby achieving fine regulation of host immune response.

However, the N protein of SARS-CoV-2 can competitively bind to E3 ubiquitin ligase TRIM25 with RIG-I, inhibit RIG-I ubiquitination and its downstream signaling pathway, and thus antagonize IFN-I production [39]. In addition, N protein can inhibit the expression of ISGs by inhibiting the phosphorylation and nuclear translocation of Signal Transducer and Activator of Transcription 1 (STAT1) and Signal Transducer and Activator of Transcription 2 (STAT2). At high doses, N protein can, on the contrary, enhance the phosphorylation and nuclear translocation of STAT1 and STAT2 and promote IFN-I reaction [93]. It is like a “two-way regulating valve”. When the content is low, it will take the lead to occupy TRIM25 and further block the signal transmission of IFN-I; when the content increases, it will activate the JAK-STAT pathway to enhance the phosphorylation of STAT1/2, thereby amplifying the expression of ISG. By precisely regulating the amplitude and duration of the host interferon response, it may help the virus secure a replicative niche, but it can also avoid excessive inflammation that leads to premature apoptosis of host cells [39].

Studies have shown that N protein can also mediate the small ubiquitin-like modifier (SUMO) modification of MAVS, a key factor of human natural immunity, by interacting with Ubc9, the only SUMO-conjugated enzyme, thereby preventing the activation of human innate immune response against COVID-19 [94]; Guo et al. constructed K18-hACE2 KI mice that stably express related proteins and established an infection model of RDPs. The results showed that the expression levels of inflammatory factors in the lung tissues of mice expressing mutant SARS-CoV-2 N(E290D) and SARS-CoV-2 N(Q349N) significantly increased, proving that the changes in amino acid positions E290 and Q349 of the SARS-CoV-2 N protein are the key factors leading to the severity of the inflammatory response after infection and being different from that of SARS-CoV [95]. These findings reveal the dual role of N protein in regulating the host innate immune response, which may be one of the pathogenesis of SARS-CoV-2, and provide a theoretical basis for the development of more effective antiviral drugs.

Notably, the M protein encoded by MERS-CoV shows a similar mechanism to SARS-CoV; that is, the M protein inhibits the binding between TRAF3 and TBK1 through direct interaction with TRAF3 molecule, thereby reducing the activation of IRF3 and its dimer formation, and ultimately leading to a decline in the expression level of IFNs [96]. However, the antagonism of coronavirus M protein to IFN signaling pathway showed virus strain specificity. For example, the M protein encoded by human coronavirus HKU1 (HCoV-HKU1) does not play an inhibitory role in the production of IFN. Compared with MERS-CoV or SARS-CoV, HCoV-HKU1 infection mostly causes only mild respiratory symptoms [97]. This phenomenon may reflect the differences in the regulation of host immune response among different coronavirus strains. These findings further highlight the complexity and specificity of coronavirus M proteins in viral pathogenicity and host immune escape mechanisms.

#### 3.2.2. Antagonism Mediated by NSPs

Coronavirus NSPs are essential enzymes and regulatory factors in viral replication and transcription. These proteins are produced by the polymeric precursors of the virus through protease cleavage and perform multiple functions in the viral replication cycle, including viral RNA synthesis, modification, and viral assembly [98]. These NSPs can significantly inhibit IFN-I production and related signal transduction pathways, and jointly inhibit the innate immune response of the host through a variety of synergistic mechanisms, helping the coronavirus to escape immune surveillance and clearance of the host [99].

NSP1 is a key immune escape factor in coronavirus, which can specifically activate intracellular endonuclease to target the 5′-untranslated region (5′-UTR) of host mRNA, a process that causes host mRNA to lose its transcriptional activity. At the same time, by binding with the 40S subunit of the ribosome, NSP1 can block the normal assembly of the 40S and 60S subunits, thus inhibiting the translation process of host mRNA [100,101], and thus blocking the host’s inherent immune response, including the generation of IFNs. Through this mechanism, NSP1 helps the virus evade the early antiviral response of the host. Intriguingly, the MERS-CoV-encoded NSP1 protein can promote endonuclease cleavage in the nucleus, and has no shearing effect on its own RNA. The specific immune antagonistic mechanism of NSP2 has not been fully elucidated, but previous studies have shown that it may be related to viral replication and may affect the signaling pathways of host cells [102]. Simultaneously, NSP2 may also regulate the host immune response by interacting with host proteins [103].

Among coronavirus NSPs, NSP3 is the largest. NSP3 contains papain-like protease (PLpro) and other domains in which PLpro exhibits significant deubiquitinating enzyme activity and modification of ubiquitin-like protein ISG15. It is worth noting that NSP3 of SARS-CoV effectively regulates the activation of the host innate immune response by inhibiting the NF-κB signaling pathway through its enzyme activity [104]. Activation of NF-κB is a multi-step process that relies on kinase IKK-mediated phosphorylation and ubiquitination modification to promote the degradation of I-κB inhibitors, followed by the release of active NF-κB and its entry into the nucleus to up-regulate IFN-associated gene expression. NSP3 of SARS-CoV specifically blocks the activation process of NF-κB signaling pathway by inhibiting the degradation of I-κB [105]. In addition, NSP3 of MERS-CoV inhibits IFN signaling mediated by RLRs such as RIG-I and MDA5, while inhibiting the activation of NF-κB [106], which is highly dependent on its protease activity.

It has been found that the combination of NSP16 and NSP10 will form a 2′-O-methyltransferase complex, which can methylate the 5′ cap of viral RNA, thereby helping viral RNA to mimic the mRNA of the host cell and evade the host’s immune surveillance, and ultimately reduce the recognition of viral RNA by the host RNA sensor [107]. In addition, NSP13 has RNA helicase activity, which may interfere with viral RNA recognition and signal transduction [108], while NSP14 has N7-methyltransferase and exonuclease activities, which can inhibit host immune response by modifying viral RNA [109]. The synergistic effect of NSP13 and NSP14 can jointly interfere with the activation of host RNA sensors and achieve the effect of antagonizing innate immunity. Furthermore, in related studies on animal models, the non-redundant immunosuppressive roles of NSP2, NSP5, etc., in the body have also been verified. For instance, Li et al. discovered that the NSP2 of porcine reproductive and respiratory syndrome virus (PRRSV) interacts with the host adaptor protein SH3KBP1 through its five atypical proline–arginine motifs and promotes its autophagic degradation, thereby antagonizing the host’s innate immunity. Moreover, the replication ability and the ability to antagonize the interferon response of the recombinant virus lacking the key motifs were weakened [110]. Wu et al. pointed out that the NSP5 of PDCoV can cleave the host protein POLDIP3 to antagonize the host’s antiviral response. This cleavage phenomenon was confirmed in SPF piglets infected with PDCoV, and the NSP5 of PEDV, porcine TGEV, and SARS-CoV-2 also have similar functions [111]. To sum up, these NSPs work together to inhibit the innate immune response of the host through a variety of mechanisms, helping the coronavirus to evade the immune surveillance and clearance of the host. Therefore, the study of the function of these proteins is of great significance for understanding the pathogenic mechanism of coronavirus and developing new antiviral treatment strategies [112].

#### 3.2.3. Antagonism Mediated by Accessory Proteins

Accessory proteins encoded by coronavirus are also essential in the viral life cycle, especially in regulating host immune response and virus pathogenicity. The accessory proteins encoded by SARS-CoV-2, SARS-CoV and MERS-CoV, such as ORF3b, ORF6, ORF4a, ORF4b and ORF5, show significant antagonistic activity against IFNs [113,114]. These helper proteins inhibit IFN signaling through a variety of mechanisms, including blocking IFN production, inhibiting activation of the IFN signaling pathway, and blocking activation or nuclear translocation of transcription factors such as IRF3 and NF-κB [87,96]. Specifically, ORF3b inhibits RIG-I and MAVS-mediated IFN production. ORF6 inhibits IFN signaling by preventing STAT1 from binding to karyopherin beta 1 (KPNB1) [115]. It has been shown that ORF4a of MERS-CoV may be able to interact with the dsRNA-binding protein PKR-activating protein (PACT) to inhibit PACT-mediated RIG-I/MDA5 activation, and ORF4b may further inhibit IFN production through interaction with TBK1 [116], but the specific mechanism is unclear and needs further investigation.

In addition, coronavirus accessory proteins such as ORF9b disrupt Hsp90/TOM70 interactions by interacting with the TOM70 complex on the mitochondrial membrane surface of host cells, thereby preventing transmission of antiviral innate immune signals [117]. By hijacking the nuclear pore complex, especially the interaction with the Rae1-Nup98 complex, ORF6 inhibits the material transport between nucleus and cytoplasm, impeding the extranuclear transport of mRNA and the entry of antiviral signaling molecules such as STAT1 into the nucleus, thus effectively inhibiting the host’s antiviral natural immune response [117,118].

Other studies have shown that ORF8 of SARS-CoV-2 can remodel the endoplasmic reticulum (ER), and accumulate in the ER cavity to cause ER stress in host cells through escape degradation by forming disulfide bond complexes with sulfyl oxidoreductase in the ER [119]. This mechanism is of great significance for understanding the pathogenicity and high infectivity of the novel coronavirus. In addition, ORF8 has also been found to have phase separation properties, by interacting with autophagy protein p62 and forming phase separation, hijacking ER autophagy receptors FAM134 and ATL3, preventing ER autophagy from occurring [120], thereby promoting viral replication. At the same time, NSPs ORF3a and ORF3b have also been shown to promote the assembly and release of SARS-CoV-2 pseudoviral particles [121], induce host inflammatory response and inhibit host IFN expression, providing new targets for the development of potential drugs.

To summarize, accessory proteins of coronavirus antagonize the host immune system through a variety of mechanisms, including inhibition of IFN signaling pathway, obstruction of nuclear translocation of antiviral signaling molecules, and disruption of host natural immune signaling [87], which play a key role in virus pathogenicity and host pathological processes. However, the current research on the function of these accessory proteins and the interaction mechanism between them and the host is not sufficient, and further exploration is needed in the future to provide more effective strategies for the treatment and prevention of coronavirus.

## 4. Conclusions

In recent years, coronaviruses have caused large-scale outbreaks worldwide three times, among which SARS-CoV broke out in 2003, MERS-CoV since 2012, and SARS-CoV-2 since 2019, with SARS-CoV-2 being the most infectious. As a newly infectious pathogen, the host, infectious source and pathogenic mechanism of SARS-CoV-2 are still to be further studied. Virus-encoded proteins can interfere with key pathways of innate immunity, such as RIG-1, NF-κB, etc., by binding or deubiquitination with key molecules in these pathways, block signal transmission and inhibit the release of IFN-I, and then affect the JAK-STAT pathway downstream of IFN-I and the expression level of ISGs, weakening the antiviral effect of IFN-I. In addition, SARS-CoV-2 may use immune escape mechanisms to replicate in the host during the initial two-week recessive infection period, eventually leading to disease outbreak. The immune escape mechanism of the virus and its interference with the acquired immune system, such as the depletion of immune cells or the induction of autoimmune attack, are important components of the pathogenicity of SARS-CoV-2. In the face of the rapid mutation of SARS-CoV-2, vaccination became the most effective preventive measure, and T-cell vaccines in particular showed greater adaptability and longer lasting immune memory. At present, in addition to the development of a variety of SARS-CoV-2 vaccines, prevention can also focus on strengthening the body’s natural immunity against coronavirus. Moreover, nanoparticles that display the conserved epitopes of pre-stored neutralizing antibodies have become a novel broad-spectrum candidate nanovaccine against COVID-19.

Considering that the genome sequence similarity between SARS-CoV-2 and SARS-CoV is as high as 79.5%, and their infection symptoms are also very similar, the body immune disorder and virus immune evasion are considered to be the main pathogenic mechanism of SARS-CoV-2. Therefore, in addition to the role of antiviral therapy, moderate immune regulation is essential to improve the prognosis and reduce the mortality of severe patients. Abnormal cytokine levels and the accumulation of large amounts of immune cells and interstitial fluid in the lungs of SARS-CoV-2 patients lead to severe respiratory obstruction and lung injury. In the future, research on coronaviruses should focus on two main aspects: mechanism analysis and clinical translation. The following contents should be prioritized for advancement: At the mechanism level, a computational model for the co-evolution of the virus and the host can be constructed, integrating multi-omics and epidemiological data to predict immune escape mutations; using multi-omics techniques, the severe immune characteristics can be systematically analyzed, key inflammatory pathways and cell subpopulations can be identified to discover precise intervention targets, etc. At the translational level, an evaluation platform for drug re-screening and combined therapy should be established, combined with artificial intelligence-assisted prediction, to evaluate the synergistic effects of antiviral drugs and immunomodulators from multiple perspectives, and promote the formulation of individualized treatment plans. The systematic advancement of these directions will build a complete research system from basic mechanisms to clinical applications, providing crucial scientific support for responding to current and future emerging viral infectious diseases [122,123].

In conclusion, coronaviruses (especially SARS-CoV-2) interfere with the host immune response through complex mechanisms, which not only affects the pathogenicity of the virus, but also poses a challenge to the development of vaccines and drugs. In the face of constant mutation of the virus, the in-depth study of the pathogenic mechanisms, the exploration of effective immune regulation strategies, and the development of virus-specific vaccines and drugs are the common goals of the global public health field.

## Figures and Tables

**Table 1:** Comparative antiviral functions of distinct PRRs.

Types of PRRs	Localization	Functional Characteristics	Antiviral Activity
TLRs	Primarily localized to the cell membrane and endosomal membrane.	Capable of recognizing conserved PAMPs such as viral dsRNA, ssRNA, and DNA.	TLR engagement initiates signal transduction that drives IFN-β and other inflammatory cytokine production, thereby restricting viral replication and dissemination.
RLRs	Cytoplasm	Specifically recognizing 5′-triphosphorylated RNA, a hallmark of replication intermediates in many RNA viruses.	RIG-I and MDA5 detect viral RNA to trigger IRF3 and NF-κB signaling cascades, thereby driving the production of IFN-I.
NLRs	Cytoplasm	Capable of sensing intracellular viral components and danger-associated molecular patterns (DAMPs), especially those signals that cause damage to organelles.	NLRC4 and NLRP3 drive inflammasome assembly and activation.
CLRs	Plasma membrane	Recognizing glycosylated pathogen components.	Members of the CLR family, DC-SIGN and Langerin, bind HIV virions and other viral particles, thereby modulating viral uptake and subsequent processing.
ALRs	Cytoplasm	Specifically recognizes intracellular dsDNA	Upon DNA virus infection, AIM2 nucleates the inflammasome by recruiting ASC, thereby activating caspase-1 and driving the maturation and secretion of IL-1β and IL-18.
cGLRs	Cytoplasm	Capable of sensing aberrantly localized cytoplasmic DNA	Upon sensing viral DNA, cGAS catalyzes the synthesis of the cyclic dinucleotide 2′,3′-cGAMP, which functions as a second messenger to activate STING and trigger IFNs production

**Table 2:** Comparative overview of the distinct immune-evasion strategies employed by coronaviruses.

Diverse Array of Strategies to Evade Immune Surveillance	Mechanisms	Evasion Kinetics	Countermeasures
Downregulation of MHC-I expression	Down-regulation of MHC-I expression is achieved through ORF6-mediated inhibition of NLRC5, ORF8-triggered autophagic degradation, and interference with signaling cascades	Down-regulation of MHC-I molecules constitutes a broadly employed immune-evasion strategy that impinges on multiple immune cell populations, most notably by crippling cytotoxic T-cell function through direct interference with host immune-signaling pathways	Blocking ORF6/ORF8 activity rescues MHC-I expression, and targeting host receptors or signaling hubs curtails viral replication and dissemination.
Immune evasion through dynamic RBD shielding	Conformational masking of the RBD cloaks key neutralizing epitopes, attenuating antibody engagement.	Dynamic remodeling of the RBD governs virus–host receptor engagement, modulates neutralizing antibody potency, and underpins humoral immune evasion.	Investigating the conformational dynamics of the RBD and designing broadly neutralizing antibodies and vaccines capable of recognizing multiple RBD conformations to elicit immune responses focused on these conserved epitopes.
Exploitation of host protease-mediated activation	Certain viral proteins require proteolytic activation by host cell proteases, a step that viruses exploit to orchestrate their life cycle—governing entry, replication, and assembly.	Protease-mediated activation by host enzymes refers to the viral exploitation of endogenous proteases to facilitate viral entry, replication, and dissemination within the host.	Development of protease inhibitors or pharmacologic modulation of the corresponding signaling cascades represents a promising antiviral target space.
Evasion of NKG2D-mediated cytotoxic immunity	Virus-encoded ORF6 down-regulates the NKG2D ligands MIC-A/B, thereby evading NK-cell-mediated immune surveillance and diminishing damage of infected cells.	Virus evades NKG2D-mediated immunity by transcriptionally down-regulating NKG2D ligands, thereby depriving NK cells of their activating signal and attenuating cytotoxic activity.	Monitor the function of NK cells and the expression of MIC-A/B, and identify and intervene in virus escape at an early stage; Alternatively, 7C6 monoclonal antibodies can be used to inhibit the shedding of MIC-A/B, enhance the antiviral efficacy to improve the ability to clear infected cells.

## Data Availability

No new data were created or analyzed in this study. Data sharing is not applicable to this article.
